# Subliminal and Supraliminal Processing of Facial Expression of Emotions: Brain Oscillation in the Left/Right Frontal Area

**DOI:** 10.3390/brainsci2020085

**Published:** 2012-03-26

**Authors:** Michela Balconi, Chiara Ferrari

**Affiliations:** Laboratory of Cognitive Psychology, Department of Psychology, Catholic University of Milan, Largo Gemelli, 1, 20123 Milan, Italy; E-Mail: chiara.ferrari.uni@gmail.com

**Keywords:** emotions, subliminal, brain oscillation, facial expression

## Abstract

The unconscious effects of an emotional stimulus have been highlighted by a vast amount of research, whereover it remains questionable whether it is possible to assign a specific function to cortical brain oscillations in the unconscious perception of facial expressions of emotions. Alpha band variation was monitored within the right- and left-cortical side when subjects consciously (supraliminal stimulation) or unconsciously (subliminal stimulation) processed facial patterns. Twenty subjects looked at six facial expressions of emotions (anger, fear, surprise, disgust, happiness, sadness, and neutral) under two different conditions: supraliminal (200 ms) *vs.* subliminal (30 ms) stimulation (140 target-mask pairs for each condition). The results showed that conscious/unconscious processing and the significance of the stimulus can modulate the alpha power. Moreover, it was found that there was an increased right frontal activity for negative emotions *vs.* an increased left response for positive emotion. The significance of facial expressions was adduced to elucidate cortical different responses to emotional types.

## 1. Introduction

### 1.1. Brain Activity and Emotional Facial Expression

The present paper intends to explore the left/right frontal brain oscillation modulation in response to different types of facial expression of emotions. Moreover, we intend to investigate the effect of supraliminal *vs.* subliminal stimulation and the direct relationship between conscious/unconscious perception and frontal brain activity.

Indeed, faces are a critically important source of social information and it appears that we are biologically prepared to perceive and respond to faces in a unique manner [1]. Brain areas generally involved in evaluation of the emotional and motivational significance of facial expressions appear to be mediated by the amygdala and orbitofrontal cortex, while structures such as the anterior cingulate, prefrontal cortex and somatosensory areas are linked to conscious representation of emotional facial expression for strategic control of thought and action [2,3]. It has been shown that the affective information contained in facial expression is perceived involuntarily [4,5], and it is able to automatically constrict the focus of attention. Considering the critical social relevance of emotional facial expressions, it is not surprising that the emotions displayed in facial expression can be perceived even when subjects have no conscious experience of perceiving them. 

Brain response to general emotional stimuli has been investigated in several studies, and frequency band variations were found to be a powerful tool to analyze the cognitive processes related to emotion comprehension [6,7,8,9,10,11]. In regard to alpha frequency (7–12 Hz), Klimesch *et al.* (1998) [12] reported that alpha frequency (in particular lower-1 alpha) desynchronizes in response to a presented warning stimulus. Some other studies have examined the alpha frequency band of the EEG and have revealed that this band can uncover cortical correlates of relatively small differences in emotional type processing [13]. Nevertheless, it was found that an anterior asymmetry in alpha reduction, explains the correlation of changes with the individual affective state [14,15,16]. 

Moreover, alpha activity in response to emotional cues has been related to the lateralization effect, and this fact makes it an interesting index to be used to test the hemispheric differences also in response to facial expression of emotions. Previous EEG research has confirmed what is stated by the valence hypothesis: relative increase of left hemisphere activity is found with positive emotional stimuli [17,18]. More recently, the approach-withdrawal model of emotion regulation posits that emotional behaviours are associated with a balance of activity in the left and right frontal brain areas that can be explained by an asymmetry measurement [19]. Resting frontal EEG asymmetry has been hypothesized to relate to appetitive (approach-related) and aversive (withdrawal-related) emotions, with heightened approach tendencies reflected in left-frontal activity and heightened withdrawal tendencies reflected in relative right-frontal activity [20]. Subjects with relatively less left- than right-frontal activity exhibit larger negative affective responses to negative emotions and smaller positive affective responses to positive emotions [21].

Some interesting results have been collected as a function of specific emotional patterns, showing a localized frontal area response to each emotional cue. In particular, sadness was correlated positively with right alpha power and negatively with left alpha power, whereas happiness was mainly related to left-side activation [22]. For other emotions, such as anger, results were more heterogeneous. More generally, lateralized electrophysiological parameters (decreased alpha power EEG), measured during the recollection of events associated with anger, increased within the right hemisphere [18]. 

Different explicative models were proposed, in order to justify this lateralization effect. The right hemisphere model supposes that the right side is specialized for the perception, expression, and experience of emotion, regardless of the valence (positive or negative) of the emotional content [23,24]. Specifically, regarding the perception of emotion, recent studies on faces have demonstrated left visual field superiority (right hemisphere) for discriminating emotional faces [25,26]. Around the expression of emotions, a facial asymmetry was found, with a more expressive left side (right-controlled) seen during emotion expression [27,28]. Also a reduced ability was identified for facial emotional expression in the case of right-hemisphere damage. Brain damage studies have confirmed this effect, showing that patients with right-hemisphere lesions performed worse than patients with left-hemisphere lesions in recognizing facial expressions [25,29]. Moreover, ERP and fMRI studies supported the hypothesis of right hemisphere specialization for the processing of facial emotions [30,31,32]. 

Nevertheless, alternative hypotheses were recently formulated on the lateralization effect, which offered different explanations of hemispheric differences. The valence model supposes that, as opposed to the right-hemisphere hypothesis, cortical differences between the two hemispheres are attributable to positive *vs.* negative valence of emotions [33,34]. In general, this model was tested for expression and perception of emotions, as well as for emotional experience. Based on this approach, the right hemisphere is specialized for negative emotions and the left hemisphere for positive emotions. 

More recently, the approach-withdrawal model of emotion regulation posits that emotional behaviours are associated with a balance of activity in left and right frontal brain areas that can be explained by an asymmetry measurement [19,35,36]. Resting frontal EEG asymmetry has been hypothesized to relate to appetitive (approach-related) and aversive (withdrawal-related) motivation and emotion. 

However, some contrasting results remain to be explained, mainly about the significance of each emotion with respect to its functional value that is the role it has for the subjective motivational system. Secondly, the significance that these results on alpha band have for the specific domain of facial expressions of emotion needs to be demonstrated. In fact, facial expressions are an important key to explain the emotional situation and, consequently, they can produce different reactions in a viewer. As a whole, the significance of emotional expressions for the subject (in terms of their high/low averseness, valence and coping potential related to the corresponding emotion) should influence both the physiological and cognitive level, with interesting correspondence on EEG modulation. It has been assumed that emotional expressions are distributed along a continuum as a function of the motivational significance of the emotional cue in terms of averseness (from higher to lower) and hedonic value (from negative to positive) and coping potential [37,38,39]. According to this assumption, the “functional model” of emotional expression supposes that people adopt behaviour that is functional to their coping activity [40,41]. Coping activity determines the significance of emotional situations, since it is able to orient the subject’s behaviour as a function of the individual expectancies about successfully acting to alter the situation/external context. In fact, whereas some negative emotional expressions, such as anger and sadness, are generated by negative, aversive situations, coping potential may introduce some differences in subjective response as a function of how people appraise their ability to cope with aversive and withdrawn situations [42,43,44]. From this perspective, anger may be appraised as a negative but also an active emotion that arouses approach motivation. In the present research this appraisal process will be tested by using agreement ratings to make sure that subjects all perceived the facial stimuli in the same way.

### 1.2. The Role of Consciousness: Subliminal/Supraliminal Stimulation Effect in Face Processing

The effect induced by perceived but not consciously elaborated emotional stimuli has been critique for a great amount of neuropsychological research, on both normal and pathological subjects [45,46,47,48,49,50,51]. More generally, facial expressions of emotion are considered unique in their ability to orient the subjective cognitive resources, even if people are unable to process information consciously. Secondly, it has been hypothesized that subjects are able to assign a semantic value to the emotional content of faces even in an unaware condition [6,52,53]. An obvious and well-known example of unconscious perception of emotion is the subliminal stimulation effect. This phenomenon has been studied in a limited number of cases [46,54]. Animal studies suggest that fear-related responses are elicited by a direct subcortical pathway from the thalamus directly to the amygdala, allowing emotion (and specifically threat) to be processed automatically as well as outside awareness. In humans, evidence for the unconscious perception of a masked face has been revealed in terms of subjective reports [55], autonomic reaction [56], brain imaging measures [57], as well as EEG and ERPs [58,59]. In addition, unconscious stimulation has been shown to be sensitive to the emotional content of the stimuli, as revealed by different behavioural and physiological measures [60]. 

However, although the existence of the unconscious effect has been accepted, the question concerning its importance for emotional decoding is still open. Only a limited number of studies has explored the significance of conscious *vs.* unconscious face comprehension, based on priming effect or subliminal stimulation [6,61,62]. The present research aims to compare these distinct processes, taking into account the effect the emotional type may have when conscious or unconscious comprehension is activated.

Another useful measure to analyze conscious and unconscious perception of faces is the masking procedure. By low intensity and brief exposure, a target stimulus can be made unrecognizable when another stimulus is presented simultaneously, shortly before (forward masking), or shortly after (backward masking) [63]. This paradigm is used to investigate below awareness response to emotional perception in which facial expressions are followed immediately by a masking face. Evidence for the unconscious perception of masked faces has been revealed in terms of subjective reports, autonomic activity, and functional brain imaging measures. 

## 2. Objectives and Hypotheses

Since asymmetrical electroencephalogram (EEG) alpha activity over anterior regions of the scalp predicts a variety of measures of interest for emotion research [64], in the present work we intend to analyze the modulation of this frequency band. Our goal was to analyze the modulation effect of EEG as a function of the different emotional types. An ample range of emotions was used in this study, in order to test the subjective response to different facial expressions, valenced as positive or negative, and more or less aversive with regards to their content. We paid attention that, varying the level of averseness and valence of each facial expression may induce different responses in the left and right hemispheres. In line with the functional model, frontal responses tended to be modulated by emotional type, with a left prevalence for positive approach expressions *vs.* negative withdrawal faces and *vice versa* for the right hemisphere. Specifically, happiness should induce a higher left side response than negative and aversive emotions such as anger, fear, surprise and sadness. The latter, conversely, should be better represented by a more right side activation.

The second goal was to explore significant resemblance between the conscious and unconscious process, since previous research has found a general qualitative similarity between the two conditions. We aimed to analyze to what extent the two processes can be analogous, specifically with respect to the semantic value of unconscious face detection. Specifically we paid attention to the emotional type effect previously stated which could be revealed in both conscious and unconscious processing, with distinct response related to the valence and arousal features of the face. Thus, the left and right lateralization effect for respectively positive and negative stimuli is expected to be found in the supraliminal and subliminal condition. These effects should be supported by an analogous left and right cortical response for alpha as a function of positive *vs.* negative emotions and high *vs.* low arousing patterns. 

## 3. Method

### 3.1. Subject

Twenty healthy volunteers took part in the study (ten women, age range 19–27, mean = 23.91, SD = 1.60). They were all right-handed and with normal or corrected-to-normal visual acuity. They gave informed written consent for participating in the study.

### 3.2. Stimulus Material and Procedure

Stimulus materials were taken from the set of pictures of Ekman and Friesen (1976) [65]. They were black and white pictures of an actor, presenting respectively a happy, sad, angry, fearful, disgusted, surprised and neutral face.

Subjects were seated comfortably in a moderately lit room with the monitor screen positioned approximately 100 cm in front of their eyes. Pictures were presented in a randomised order in the centre of a computer monitor, with a horizontal angle of 4° and a vertical angle of 6° (STIM 4.2 software). During the examination, participants were requested to minimize blinking. They were required to observe the stimulus during EEG recording (passive task). In the subliminal condition it was emphasized that sometimes the target face would be difficult to see, but the subjects were requested to concentrate as best they could on stimulus, since they would be asked questions about these stimuli after the EEG recording. An explicit response to the emotional features of the stimulus was not required. This was done for three main reasons: to assure a real passive task (implicit elaboration of emotions); not to cause them to be more attentive to the emotional stimuli than the neutral ones; not to introduce an unequal condition between conscious and unconscious stimulation. Prior to recording EEG, subjects were familiarized with the overall procedure, where every subject saw in a random order all the emotional stimuli presented in the successive experimental sessions (14 trials, each expression type repeated twice).

### 3.3. Backward Masking Procedure

During the experiment we used a backward masking procedure. Each facial stimulus (target) was presented for either 30 (low exposure) or 200 (high exposure) ms, followed by another face presented for 200 ms (masking stimulus, a neutral face). Inter-stimulus interval was 200 or 30 ms respectively for supraliminal *vs.* subliminal condition [66,67,68]. Thus the trail duration might vary between 260 and 600 ms as the function of stimulus condition. Inter-trial interval was 2 s. 

In total there were 140 target-mask pairs in each threshold condition (each expression type was presented twenty times for condition). Stimuli were totally randomized across emotional type. Different blocks of stimulation were performed distinctly for supraliminal and subliminal condition and they were varied across-subjects with respect to their order. The short stimulus presentation in the subliminal condition prevents the subjects having a clear cognition of the stimulus. In the current study we employed an objective threshold for the masked condition. It was defined by an identification procedure, the case where stimulus is perceived by the subject no more than in 50% of the cases [68,69]. According to signal detection theory (SDT [70]), when detection threshold sensitivity is at chance (*d*’ = 0), it is unlikely that there is conscious awareness of the stimulus.

The *post-hoc* briefing was used to test that the subjects were unable to detect target stimulus in the subliminal condition. Target-mask pairs were randomized across condition and emotional type. Specifically, we presented each emotional expression two times, for each condition (supraliminal and subliminal) for a total of 28 target-mask pairs (for details on procedure see previous). After each pair, subjects were requested to identify the target stimulus, by a categorization task (to recognize the specific emotion represented by the face). No more than chance recognition was found for the pre-attentive condition. The accuracy of facial expression discrimination in the subliminal trials was 50.4%, and it did not differ from chance (*t* < 1). On the contrary, supraliminal elaboration produced a correct recognition for each emotional category (response accuracy = 98%), and above chance, *t*(20) = 31.23, *p* < 0.001.

Secondly, the subjects were asked to analyze the stimuli viewed after the experimental section, in order to check for the emotional comprehension of facial expressions. The target stimuli were presented to the subjects in a supraliminal condition (200 ms) and in a random order. They evaluated the emotional significance of each expression by a categorization task. The emotions were correctly recognized by the subjects (happiness, M = 95%; anger, M = 97%, sadness, M = 91%; fear, M = 95%; disgust, M = 96%; surprise, M = 97%; neutral, M = 92%). Subjects were also asked to rate their agreement of judgement. The seven emotional categories were correctly recognized. Specifically a high judgement agreement was done for happy (on a five-point Likert scale: M = 4.53; SD = 0.25), sad (M = 4.48; SD = 0.23), angry (M = 4.60; SD = 0.33), fearful (M = 4.51; SD = 0.40), disgusted (M = 4.38; SD = 0.25), surprised (M = 4.39; SD = 0.34) and neutral (M = 3.90; SD = 0.28) faces. 

### 3.4. EEG Acquisition and Data Reduction

The EEG was recorded with a 32-channel DC amplifier (SYNAMPS system) and acquisition software (NEUROSCAN 4.2) (for the procedure see [37]). An ElectroCap with Ag/AgCl electrodes was used to record EEG from active scalp sites referred to earlobe (10/20 system of electrode placement). Additionally two EOG electrodes were sited on the outer side of the eyes. The data were recorded using sampling rate of 500 Hz, with a frequency band of 0.1 to 40 Hz. The impedance of recording electrodes was monitored for each subject prior to data collection and it was always below 5 kΩ. A successive regression-based eye movement correction was used. The computerised artefact rejection criterion excluded the peak-to-peak amplitude when it exceeded 50 μV. After EOG correction and visual inspection only artefact-free trials were considered (5% discarded for artefacts). Only fourteen electrodes were used for the successive analysis (four central, Fz, Cz, Pz, Oz; ten lateral, F3, F4, C3, C4, T5, T6, P3, P4, O1, O2). The digital EEG data were bandpass filtered in the 8–12 Hz frequency band (band-pass filtering 96 dB/octave rolloff, warm-up filter left and right to 100 ms). To obtain a signal proportional to the power of the EEG frequency band, the filtered signal samples were squared and successively log-transformed [71]. Successively, the data were epoched, using a time window of 900 ms, and epochs were triggered to the stimulus appearance. An average absolute power value for each electrode for each condition was calculated. An average of the pre-experimental absolute power (−200 ms) was used to determine the individual power during no stimulation. From this reference power value, individual power changes during stimulus viewing were determined as the relative stimulus-related decrease or increase. For the successive statistical analysis only F3/F4 electrode sites were used to compare frontal left and right activation. Not less than eighteen epochs for each condition and emotional type were considered to obtain the average.

## 4. Results

The dependent variable of alpha power was entered into a three-way ANOVA using the following repeated factors: condition (2: supraliminal/subliminal) × side (2: right/left) × emotion (7). Type I errors associated with inhomogeneity of variance were controlled by decreasing the degrees of freedom using the Greenhouse-Geiser epsilon correction. 

Significant effects were found for emotion and emotion × side (Table 1 and Table 2).

**Table 1:** Mean power of alpha in response to each emotion, side and condition effects.

	Right (Supra-)		center (Supra-)		Right (Subli-)		center (Subli-)	
	M	(SD)	M	(*SD*)	M	(*SD*)	M	(SD)
anger	6.58 ^a^	1.28	8.08	1.10	7.34	1.47	9.56	1.56
fear	7.05	1.30	8.05	1.24	7.60	1.27	9.77	1.09
disgust	7.09	1.05	8.20	0.89	8.73	1.40	9.46	0.98
surprise	7.10	1.27	8.43	0.43	7.35	0.83	9.30	0.97
happiness	8.53	1.87	6.70	1.66	12.10	1.05	8.15	1.53
sadness	10.98	1.50	10.89	1.32	11.43	1.22	9.87	1.23
neutral	12.30	1.33	12.78	0.72	12.77	1.19	11.02	1.90

a = measured in mVolt2.

**Table 2:** Main and interaction effects (only significant) for repeated measurement ANOVA (Greenhouse-Geiser epsilon correction).

Source		df	F	*p*	η^2^
emotion		6	12.43	0.001	0.40
emotion × side		6	9.61	0.001	0.35

Planned contrasts applied to the main effect of emotion showed differences between anger and sadness (F(1,19) = 8.79, *p* = 0.001, η^2^ = 0.39), and fear and sadness (F(1,19) = 6.91, *p* = 0.001, η^2^ = 0.34), with a decreased alpha power (increased activity) for anger and fear respectively. Moreover, neutral stimulus differed from the other emotions (all comparisons *p* = 0.001). In addition, simple effects (emotion × side) revealed an increased activity in the frontal right cortical side for anger as compared to happiness, sadness, and neutral faces (all comparisons *p* = 0.001); fear compared to happiness, sadness, and neutral faces (all comparisons *p* = 0.001); surprise compared with sadness, and neutral faces (all comparisons *p* = 0.001). Moreover, an increased activity was found in the left frontal area for happiness compared with anger and fear (F(1,19) = 6.78, *p* = 0.001, η^2^ = 0.33). 

Two distinct analyses were successively performed for the supraliminal and subliminal conditions. Similar effects induced by the two experimental conditions in relation to emotions and side variables were verified. 

**Figure 1:**
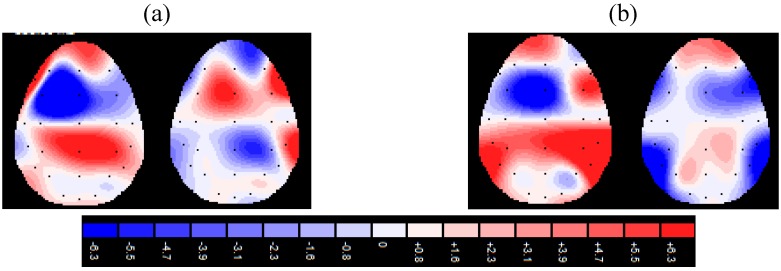
Alpha power for supraliminal (**a**) and subliminal (**b**) condition in response to positive and negative aversive facial expressions.

For the supraliminal condition, emotion and emotion x side significant effects were found (Table 3). Planned comparisons revealed a significant main effect of emotion with significant differences between anger and sadness (F(1,19) = 8.16, *p* = 0.001, η^2^ = 0.38), and fear and sadness (F(1,19) = 8.89, *p*= 0.001, η^2^ = 0.41), with an increased activity for anger and fear respectively. The simple effects (emotion × side) revealed a higher activity in the frontal right cortical side for anger as compared to happiness, sadness, and neutral faces; fear compared to happiness and neutral faces; surprise compared with sadness, and neutral faces (all comparisons *p* = 0.001). Moreover, an increased activity was found in the left frontal area for happiness compared with anger and fear (Figure 1a).

**Table 3:** Supraliminal condition. Main and interaction effects (only significant) for repeated measurement ANOVA (Greenhouse-Geiser epsilon correction).

Source		df	F	*p*	η^2^
emotion		6	10.16	0.001	0.43
emotion x side		6	9.78	0.001	0.36

For the subliminal condition, emotion and emotion × side effects were significant (Table 4). As planned comparisons revealed significant differences between anger and sadness (F(1,19) = 9.03, *p* = 0.001, η^2^ = 0.42), and fear and sadness (F(1,19) = 8.77, *p* = 0.001, η^2^ = 0.40), with a higher response for anger and fear respectively. The simple effects (emotion × side) revealed a higher response in the frontal right cortical side for anger as compared to happiness, sadness, and neutral faces; fear compared to happiness and neutral faces; surprise compared with sadness (all comparisons *p* = 0.001). Moreover, an increased activity was found in the left frontal area for happiness compared with anger and fear (Figure 1b).

**Table 4:** Subliminal condition. Main and interaction effects (only significant) for repeated measurement ANOVA (Greenhouse-Geiser epsilon correction).

Source		df	F	*p*	η^2^
emotion		6	10.09	0.001	0.42
emotion × side		6	9.33	0.001	0.37

## 5. Discussion

Three main points can explain the results found by the present research. 

First, a clear frontal cortical activity was revealed in response to emotional faces, specifically for the emotions of fear, anger, surprise, disgust, and happiness. In addition, around the lateralization effect, the right frontal area was differentially activated by facial expression types: the right anterior side was mainly responsive to the emotions of anger, fear, and surprise as compared to sadness or positive (happiness) emotions and neutral stimuli. On the contrary, happiness activated more the left than the right cortical side. Second, supraliminal *vs.* subliminal stimulation showed similar frontal modulation as a function of both the emotional types and the hemisphere effect, in line with the circumflex model of emotion comprehension. That is, the unconscious perception of facial expressions seems to be able to produce an analogous and specific frontal response to different emotional types taking into account valence and arousal features. 

The findings from EEG alpha activity lend support to increased frontal cortical activity in response to facial expressions of emotions. Previous studies have underlined the significance of frontal sites for face perception: a general frontal facilitation effect was found for facial stimulus processing rather than posterior or temporal areas [72,73]. Nevertheless no systematic comparison has been previously conducted between different types of emotional expressions. Conversely, the present research enabled demonstration of the specific frontal right/left responsiveness to differently valenced stimuli. In fact, greater right than left side activation was found for some negative emotions and a reverse tendency (left- more than right-side) was found for the positive emotion of happiness. Thus, negative emotional stimuli are able to induce a more intense response of the right hemisphere, whereas positive ones are responsible for a more accentuated left-response. 

A possible explanation for this finding may be that the right hemisphere selectively attends more to negative facial stimuli and, conversely, the left hemisphere shows increased attention toward positive faces. In fact, studies applied to normal or clinical samples have found hemispheric differences as a function of positive *vs.* negative emotions, differences that are attributable to the facility of the two hemispheres in identifying specific emotional types [25,26,28]. For example, it was found that reaction times were shorter for happy faces shown within the right visual field (left hemisphere) and sad faces presented within the left visual field (right hemisphere) [74]. Previous studies have underlined the differential impact of anger and fear on facial expressions in comparison to sadness [37,75,76].

Nevertheless, a main critical point to be discussed in the present contribution is the fact that the right-frontal prevalence was found for all the negative emotional faces but not for sadness and, partially, for surprise. This result contrasted with the previous right-side negative hypothesis based on the valence model and it is unexplained by other empirical investigations (see for example, [74]). Based on these data, a more exhaustive paradigm of analysis may be adopted in addition to the valence hypothesis, in order to explain our results, taking into account both the valence (positive *vs*. negative) and degree of averseness of the emotional stimuli (high *vs*. low). The circumflex model is able to explain the frontal-right facilitation for some emotional types, that is the negative, high arousal, aversive emotional expressions, and the frontal-right inhibition for those emotions that have a less arousing power, with a concomitantly reduced significance in producing a lateralization effect [77,78]. This model allows integration of a more exhaustive perspective, that can directly account for the specific semantic value that each emotion has in terms of arousing power and valence. Thus the circumflex model predicts that the structure of emotional expression and comprehension is related to a roughly circular order in a two-dimensional space, the axes of which could be interpreted as pleasure-displeasure and arousal-sleepiness. In particular, the two orthogonal axes allow for a clear categorization of emotion perception, subdividing the entire emotional universe as a function of arousal response produced by emotional patterns in addition to the negative/positive value they have. In general, it is possible that the higher aversive and arousing stimuli (fear, anger, disgust and surprise) may have induced a clear cortical lateralization within the right side, whereas sadness generates a less significant response in the subjects. Moreover, adopting the avoidance-approach model, we can state that emotions more directly related to avoidance (such as fear and disgust) may be more represented within the right-cortical module, whereas the approach-related emotions (such as happiness) may be mainly left-side localized. However some integrative explanations should be considered to describe other emotional types, such as sadness, that has been considered a typical avoidance-emotion by some models or an approach-emotion by other models. In the present research no exhaustive results have been found to be either in favour or against one or other of the models.

Secondly, based on the present results, similarities in processing between supraliminal and subliminal stimulation can be assessed: consistent analogies in the subliminal and supraliminal EEG alpha responses were well-founded, suggesting that similar neural activity is involved. This is a second main and innovative point of the present research, since a systematic comparison was conducted between conscious *vs.* unconscious processing taking into account the different emotional expressions as a function valence and the significance each face has with respect to the aversive *vs.* approach model. 

Thus, a first conclusion is that the frontal EEG synchronization may also index unconscious processes related to emotion comprehension. The two processes, attentive and pre-attentive, revealed ample space for similarity, since they are marked by analogous cortical activation of the frontal area. A related and important result is that conscious *vs.* unconscious comparison provides compelling evidence that emotional stimuli are perceived and semantically analyzed even when they are presented under stimulus conditions that make it impossible to have a conscious comprehension of information. It enables us to answer the semantic significance of the pre-attentive elaboration: the subjects, although unaware, can attribute an emotional value to facial expressions. In other words, information presented to subjects under subliminal condition may be processed on a high level even if the subject is not aware of this information [79]. This is in line with studies that have examined psychophysiological responses to unconscious emotional stimuli: they were effective both in capturing attention and in eliciting autonomic response (skin conductance measurements or cardiovascular indexes) or conditioned responses [80]. That said, unconscious affective stimuli may have an effect on the appraisal of conscious stimuli. The subliminal process, that appears to have a preattentive origin and that can observe stimuli that are prevented from reaching conscious recognition, could have adaptive value because it allows an immediate response to a relevant and potentially threatening stimulus, and this system can operate even prior to the conscious appraisal of the stimulus. 

An interesting effect was found for frontal brain oscillations in subliminal conditions, where different responses by the subjects were observed for some types of emotion, analogously to the supraliminal condition. The first main fact is that data also suggest an emotional specificity in sub-threshold elicited responses, which indicates that the stimuli can be positively or negatively evaluated without conscious recognition. Secondly, the direction of the differences was the same in both the attentive and pre-attentive condition, with an increased cortical response for high arousal (both positive *vs.* negative) of emotional faces (like happiness, fear, anger, disgust and surprise). The absence of statistical differences between conditions as a function of emotion induces the inference of a similar frontal response for the two stimulation types, with analogous differences between the emotional expressions.

Our results support the functional significance of facial expressions, since facial expressions seem to be intrinsically valenced, possibly serving as simple releasing mechanisms for approach or avoidance response. Moreover, a substantial amount of research has established that unconsciously perceived facial expressions elicit emotional responses that include various forms of physiological arousal. In particular, when a more salient facial expression is perceived without awareness, subjects show a pattern of physiological arousal that includes, amongst others, larger skin conductance response [47], ERP measurements [6,68] and brain oscillation responses [81].

Moreover, our results enabled us to extend the range of emotions to be compared and to explore in detail the functional value of facial expressions as well as in the case of an unconscious perception. It is assumed that, as a function of salience, stronger for negative or high arousal stimuli, each facial expression has a specific site along a continuum, a fact that was revealed by the increased frontal activity. From an evolutionary perspective, some emotions appear to be prominent as a human safeguard [82], since they facilitate the survival of the species. The immediate and appropriate response to these emotionally salient stimuli confers on them an adaptive value: amongst others, more negative and threatening facial stimuli (such as anger or fear) may evoke greater arousal than unthreatening stimuli,a fact that may produce specific physiological and cognitive reactions, marked by increased cortical activation.

## 6. Conclusions

In conclusion, the EEG measures showed a broad sensitivity to the motivational significance of faces, varying as a function of the degree of negativity/positivity and averseness power (high/low) of the emotional cues [58]. From this perspective, the significance of emotions offers a valid explanatory hypothesis to the hemispheric differences as revealed in the present study. Left- and right-side activation was also demonstrated in response to positive and negative faces respectively, even if it was mediated by the emotional significance of facial expressions in terms of their approach *vs.* avoidance attitude. In addition, both conscious and unconscious perception of emotional facial cues may have an effect in modulating cortical brain oscillations, showing analogous patterns of response for stimuli that are presented under or over the attentive threshold. The existence of similar cortical pathways able to synchronize the frontal brain oscillations may suggest an important “semantic power” of the unconscious perception, although less differentiated and detailed in comparison with the conscious perception. 

Nevertheless some main points should be explored in future research. Firstly, a direct comparison between frontal area and other cortical sites should be conducted. Secondly, the contribution of other frequency bands should be investigated taking into account the significance they have for facial expressions in relation to specific brain localization where these frequency bands appear to be distributed. Thirdly, a more direct manipulation check on the subjective evaluation of emotional response to facial expression should be included, in order to evaluate the contribution of the valence and approach/withdrawal model for the lateralization of face comprehension.
